# Editorial: Spotlight on Europe - chemical sciences 2023

**DOI:** 10.3389/fchem.2024.1446943

**Published:** 2024-07-10

**Authors:** Clara S. B. Gomes, Alessandro Contini, Alessandro Pratesi, Pellegrino Musto, Félix Zamora, Etienne Brachet, Tony D. James

**Affiliations:** ^1^ LAQV-REQUIMTE, NOVA School of Science and Technology (NOVA FCT), NOVA University of Lisbon, Caparica, Portugal; ^2^ Dipartimento di Scienze Farmaceutiche-Sezione di Chimica Generale e Organica “Alessandro Marchesini”, Università degli Studi di Milano, Milano, Italy; ^3^ Department of Chemistry and Industrial Chemistry, University of Pisa, Pisa, Italy; ^4^ Institute for Polymers, Composites and Biomaterials, National Research Council of Italy, Pozzuoli, Italy; ^5^ Departamento de Química Inorgánica and Condensed Matter Physics Center (IFIMAC), Universidad Autónoma de Madrid, Madrid, Spain; ^6^ Faculté de Pharmacie de Paris, Université Paris Cité, UMR-CNRS CiTCoM, Paris, France; ^7^ Department of Chemistry, University of Bath, Bath, United Kingdom

**Keywords:** chemical sciences, peptide sub-microfibers, crystallography, catalytic cycle, photoluminesce, lab-on-a-molecule

We are pleased to present the Research Topic “*Spotlight on Europe-Chemical Sciences 2023*”in Frontiers in Chemistry. This Research Topic aims to celebrate the exceptional contributions of European-based researchers across the vast and dynamic field of chemistry.

Frontiers in Chemistry, a multidisciplinary gold open-access journal, has meticulously curated this Research Topic to highlight the high-quality research and innovative developments spearheaded by internationally recognized European scientists. We aim to showcase the pioneering work that addresses significant theoretical, experimental, and methodological challenges with far-reaching applications to pressing scientific and societal problems.

European research in chemistry plays a pivotal role in driving scientific innovation and addressing global challenges. From advancing sustainable energy solutions to developing new materials and pharmaceuticals, European chemists are at the forefront of scientific breakthroughs that improve our quality of life and protect our environment. The scientific works presented in this Research Topic exemplify the critical contributions of European researchers to the global scientific community.

The “*Spotlight on Europe-Chemical Sciences 2023”* Research Topic underscores European scientists’ vibrant and influential role in advancing the frontiers of chemical sciences. By presenting a diverse array of research topics, this Research Topic highlights the breadth and depth of European expertise and innovation in chemistry.

We congratulate all authors whose remarkable research is featured in this Research Topic. Their work not only exemplifies scientific excellence but also inspires future innovations in the chemical sciences. This Research Topic features four original articles and one mini-review written by researchers from different countries, including Portugal, Brazil, Switzerland, Spain, China, Italy, Sweden, and Malta. These articles illustrate the wide range of chemical research and its applications.

The issue includes a fantastic contribution from A Goncalves Carvalho Dias et al., who have described a methodology for preparing peptide sub-microfibres obtained by solution blow spinning. Solvent modulation was highlighted as a process for obtaining peptide sub-microfibres with different morphologies and diameters. The production of these fibres was aided by using a synthetic polymer in two different solvent conditions. Specifically, peptides in acetic acid self-assemble into distinct fibers around PVP, whereas mixed PVP-peptide fibers are formed in isopropanol. The ability to dissolve PVP fibers without damaging peptide integrity further highlights the versatility of this method. Thus, this work unlocks a new approach to producing peptide-based fibres for a variety of bioengineering applications.

The crystal structure and cation-anion interactions of a sulfonimide salt, namely, the potassium (difluoromethanesulfonyl) (trifluoromethanesulfonyl) imide {K[N(SO_2_CF_2_H)(SO_2_CF_3_)], KDFTFSI}, were established by Sánchez-Diez et al. using single-crystal X-ray diffraction and DFT calculations. Sulfonimide salts are indeed of great interest for battery applications. KDFTFSI shows a higher bond valence for K···O interactions than the conventional potassium bis (trifluoromethanesulfonyl) imide salt, indicating stronger cation-anion interactions due to hydrogen substitution. Additionally, DFT calculations highlight the higher stability of the *cis* conformation in both DFTFSI^−^ and TFSI^−^, with H-bonding increasing the energy gap for *trans*-*cis* conversion in DFTFSI. This work has provided a better understanding of the structure-property relationships of hydrogenated sulfonimide anions, enabling the next-generation of anions to be designed for battery research.

In another outstanding contribution, Cirri et al. describe an unprecedented catalytic cycle based on palladium and arsenic to hydrate nitriles to the corresponding amides. The reaction occurs under mild aerobic conditions, including neutral pH and moderate heating, using commercially available catalysts. The versatility of the catalytic cycle was demonstrated across various nitriles, including aliphatic and aromatic systems, with different ring substitutions and functional groups, and a pilot study suggested the potential for easy scale-up.

The preparation and complete characterisation of photoluminescent copper(I) iodide alkylpyridine thin films with the general formula [CuI(L)]n, where L = alkylpyridine derivatives such as 4-methylpyridine, 3-methylpyridine, 2-methylpyridine, etc., have been reported by Jamshidi et al. These films exhibit fast and reversible luminescence quenching when exposed to volatile halogen-based compounds (VHCs), making them promising sensors. The study highlights that the steric hindrance of ligands affects the polymeric structure and stability of the films, with bulkier ligands enhancing stability. However, sensitivity to air pressure suggests a need to consider stability when using CuI films in practical applications.

The review by Magri focuses on the concept of a “lab-on-a-molecule”, where molecules capable of detecting multiple analytes, including through fluorescence, are reviewed and discussed in the context of molecular logic and multi-analyte sensing. This mini-review highlights challenges and opportunities for moving the boundaries of research in this field, even though very few molecules can act as multi-sensing chemosensors, and the majority only detect cations. In particular, the author calls for more involvement from skilled synthetic organic chemists to advance this field, emphasizing the need for more anion receptors, the adaptation of known ditopic fluorescent logic gates, the use of efficient synthetic strategies, and inspiration from fluorescent natural products.

The guest editors of this Research Topic express their gratitude to the contributors and reviewers who were instrumental in developing this Research Topic. We look forward to the discoveries and innovations that will emerge from this Research Topic, and we are confident that it will serve as a catalyst for future breakthroughs.

